# Components of Metabolic Syndrome in Youth With Classical Congenital Adrenal Hyperplasia

**DOI:** 10.3389/fendo.2022.848274

**Published:** 2022-03-24

**Authors:** Mimi S. Kim, Nicole R. Fraga, Nare Minaeian, Mitchell E. Geffner

**Affiliations:** ^1^ Center for Endocrinology, Diabetes and Metabolism, Children’s Hospital Los Angeles, Los Angeles, CA, United States; ^2^ Keck School of Medicine of University of Southern California, Los Angeles, CA, United States; ^3^ The Saban Research Institute at Children’s Hospital Los Angeles, Los Angeles, CA, United States

**Keywords:** congenital adrenal hyperplasia, cardiovascular disease risk, metabolic syndrome, pediatrics, children, adolescents, pediatric obesity

## Abstract

Classical congenital adrenal hyperplasia (CAH) due to 21-hydroxylase deficiency is the most common primary adrenal insufficiency in children, involving cortisol deficiency, hyperandrogenism, and cardiometabolic risk. Prior studies have reported that youth with classical CAH have a higher prevalence of the components of metabolic syndrome: obesity, hypertension, elevated fasting blood glucose, and dyslipidemia. Yet, the incidence of the complete metabolic syndrome itself in children and adolescents with CAH is relatively rare. Traditional cardiometabolic risk factors can surface early in children with classical CAH, and continue to present and evolve over the lifetime, although it is only recently that reports of Type 2 diabetes and adverse cardiac events have begun to surface in adults affected by this condition. The pathophysiology underlying the increased prevalence of cardiometabolic risk factors in patients with CAH is not well-understood, with disease treatments and androgen excess having been studied to date. The aim of this review is to evaluate the recent literature on traditional cardiometabolic risk factors in youth with classical CAH, and to consider non-traditional risk factors/biomarkers for subclinical atherosclerosis, inflammation, and insulin resistance. A better understanding of these traditional and non-traditional risk factors in youth with CAH could help guide treatment options and prevent the onset of metabolic syndrome in adulthood, reducing overall patient morbidity.

## Introduction

**Figure 1:**
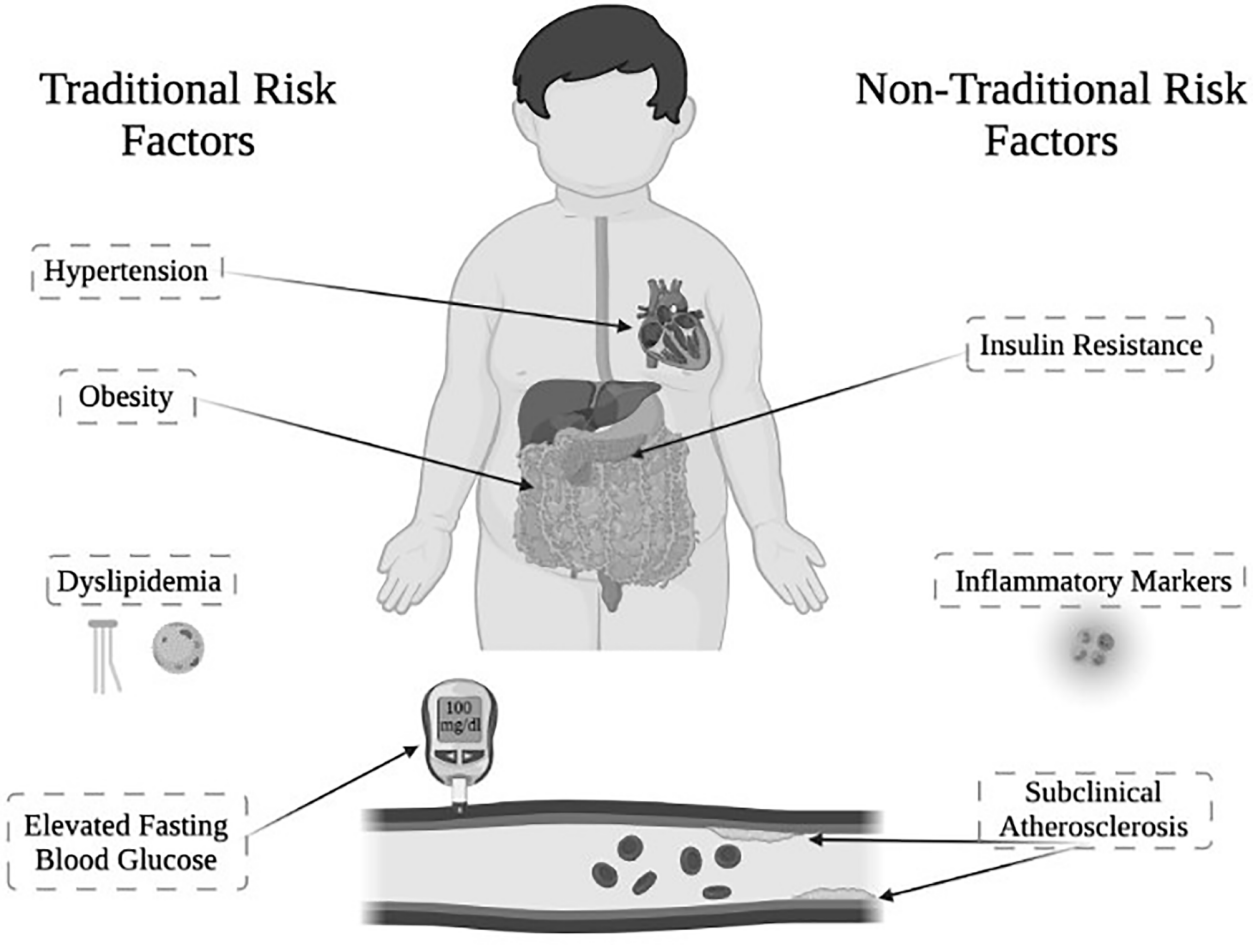
Cardiometabolic risk factors in youth with classical CAH due to 21-hydroxylase deficiency. Traditional and non-traditional cardiometabolic risk factors observed in youth with classical CAH. *Figure created with Biorender.

Classical congenital adrenal hyperplasia (CAH) due to 21-hydroxylase deficiency is the most common primary adrenal insufficiency in youth, affecting ~1 in 15,000 live births. CAH is characterized primarily by inadequate production of cortisol and aldosterone, along with overproduction of androgens (1, 2). Youth with CAH are not only affected by these hormone imbalances, but also exhibit an increased prevalence of cardiometabolic risk factors, which constitute the components of metabolic syndrome (**Figure 1**). Metabolic syndrome in youth is defined by having at least three or more of the following criteria: obesity, hypertension, elevated fasting blood glucose, and dyslipidemia (3).

Youth with classical CAH exhibit a higher prevalence of obesity across several countries (4–9) compared to their unaffected peers (10). Additionally, youth with CAH exhibit a heightened risk of hypertension (9, 11–13), and recently have been reported to have an increased prevalence of elevated fasting glucose, as well as dyslipidemia that worsens with increasing age (14).

Although youth with CAH manifest these individual components of metabolic syndrome, there have been fewer reports of the complete metabolic syndrome in youth with CAH than might be expected until recently (5, 14, 15). As a result, it would be enlightening to also consider non-traditional cardiometabolic risk factors in youth with CAH, during adolescence and to examine how these factors could potentially help to identify those patients who are at higher risk of developing metabolic syndrome. Non-traditional cardiometabolic risk factors to consider that have been studied in CAH include: subclinical atherosclerosis (16–18), inflammatory markers (19), and insulin resistance (5, 17, 19) (**Figure 1**). Importantly, a large, retrospective matched cohort study in Sweden showed that patients with CAH not only have increased prevalence of cardiometabolic risk factors, but that older adults demonstrate increased cardiovascular mortality (20), signaling a demand for further research to better understand cardiometabolic health in CAH.

In this short review, we provide an overview of current knowledge regarding the individual components of the metabolic syndrome in youth with CAH, with a focus on evidence from 2015 onwards for both traditional and non-traditional cardiometabolic risk factors (**Table 1**). As well, we discuss gaps in knowledge and areas for future research.

**Table 1:** Cardiometabolic risk factors and classical congenital adrenal hyperplasia: references from 2015 to present.

First Author Year	CAH Study Population	Age, Sex	Main Outcomes	Conclusions
Akyürek, N. 2015 (21)	N = 25	5-15 years64% Female	CAH patients had increased BMI, insulin resistance, diastolic blood pressure (DBP) and carotid intima-media thickness (cIMT). 24% of patients exhibited arterial hypertension, and 20% had nocturnal hypertension. CIMT was higher in patients with nocturnal hypertension.	Classical CAH patients exhibit subclinical cardiovascular disease (CVD) with associations with hypertension.
Falhammar, H. 2015 (20)	N = 588	0-40 years57% Female	Increased prevalence of hypertension, obesity, hyperlipidemia, and diabetes observed in CAH patients vs. controls.	CAH was associated with higher rates of cardiovascular and metabolic morbidity.
Kim, M.S. 2015 (15)	N = 28	15.6 ± 3.2 years	Visceral adipose tissue (VAT), subcutaneous adipose tissue (SAT), and VAT : SAT were higher in CAH patients vs. controls.	Increased prevalence of unfavorable abdominal fat distribution could affect CVD risk in CAH.
		54% Female		
Marra, A.M. 2015 (22)	N = 20	13.6 ± 2.5 years	CAH patients had increased BMI, waist-to-height ratio and HOMA index vs. controls, and high systolic blood pressure (SBP) and decreased workload at peak exertion.	CAH patients can exhibit decreased exercise tolerance due to subclinical cardiovascular abnormalities.
		50% Female		
Rodrigues, T.M.2015 (23)	N = 40	5-20 years	Increased cIMT was observed in CAH youth, who also presented with increased BMI and SBP compared to controls.	Increased cIMT, BMI and SBP from a young age suggests increased CVD risk in CAH.
		80% Female		
Bonfig, W. 2016 (11)	N = 716	3-18 years	Prevalence of hypertension in the study population was 12% and was more prominent in younger CAH patients.	CAH patients have increased risk for hypertension. However, the prevalence decreases with age.
Kim, M.S. 2016 (24)	N = 20	16 ± 3.3 years	Mean cIMT was correlated with serum 17-hydroxyprogesterone (17-OHP) and androstenedione levels in CAH patients. No cIMT differences observed between CAH patients and controls.	Findings suggest a link between hyperandrogenism and subclinical atherosclerosis in CAH patients.
		50% Female		
Metwalley, K.A. 2016 (25)	N = 32	13.6 ± 2.5 years	Higher levels of highly-sensitive C-reactive protein (hs-CRP) and circulating endothelial cells in CAH patients, as well as left ventricular hypertrophy and prolonged mitral deceleration time.	Children with CAH present with markers of endothelial damage, subclinical atherosclerosis and left ventricular dysfunction.
		56% Female		
Takishima, S. 2016 (26)	N = 29	Pediatric	Adiposity rebound (AR) in CAH patients occurred before the age of 4 years, which is earlier than the general Japanese population.	Lower BMI at birth is associated with earlier AR in CAH patients.
		52% Female		
Ariyawatkul, K. 2017 (27)	N = 21	15.2 ± 5.8 years	Increased waist-to-hip ratio in patients with classical CAH.	Adolescents with CAH have increased risk of visceral obesity and cardiometabolic risk factors.
		81% Female		
Mooij, C.F. 2017 (12)	N = 27	8-16 years	Elevated BMI and blood pressure observed in CAH patients, with seven patients categorized as overweight and four as obese.	Elevated BMI and blood pressure in CAH patients from a young age increases their CVD risk.
Sarafoglou, K. 2017 (7)	N = 194	≥ 2 years	Children with CAH had increased risk for early onset obesity. AR occurred earlier at 3.3 years old.	Careful monitoring of hydrocortisone dosing during early childhood is needed to prevent increased weight gain and early AR in CAH.
		52% Female		
Wierzbicka-Chimel, J. 2017 (28)	N = 19	23.7 ± 3.8 years	CAH patients had decreased flow mediated dilation (FMD), cIMT, and common femoral artery IMT (fIMT).	CAH patients on long-term glucocorticoid therapy demonstrate decreased FMD and subclinical changes in left ventricular diastolic function.
		37% Female		
Metwalley, K.A. 2018 (29)	N = 36	5-12 years	CAH patients had elevated serum homocysteine levels, thicker cIMT, and high left ventricular mass.	Elevated homocysteine levels in CAH patients suggests risk for subclinical atherosclerosis.
		72% Female		
Tamhane, S. 2018 (30)	Meta-Analysis	Pediatric and Adult	CAH patients had increased SBP, DBP, insulin resistance, and cIMT, but no evidence of morbidity or mortality due to cardiac events.	CAH patients have a high prevalence of cardiometabolic risk factors, but evidence has been lacking for actual morbidity or mortality.
Improda, N. 2019 (31)	Review Paper	Children and Adolescents	CAH patients presented with obesity, insulin resistance, hypertension, increased IMT and subclinical cardiac dysfunction from a young age.	Exposure to excess glucocorticoids, mineralocorticoids, and androgens may contribute to the development of cardiovascular changes.
Metwalley, K.A. 2019 (32)	N = 36	13.7 ± 2.4 years	CAH patients had greater epicardial fat thickness (EFT), cIMT, and left ventricular mass vs. controls.	Increased EFT suggests an increased risk of developing left ventricular dysfunction and subclinical atherosclerosis in CAH.
		69% Female		
Vijayan, R. 2019 (33)	N = 52	3-21 years	CAH patients had a higher BMI, mean DBP, and greater insulin resistance vs. controls.	CAH youth have higher CVD risk and reduced quality of life despite adequate management.
		(Median 12y)		
		73% Female		
Bhullar, G. 2020 (34)	N = 42	45.2% Female	CAH patients had earlier AR at 3.4 ± 1.3 years overall, and patients with obesity had an earlier AR vs. lean patients. Earlier AR predicted higher BMI-z during childhood, as well as increased central obesity and total body fat in adolescence.	Early AR can be used as a marker for disease severity and cardiometabolic risk in youth with classical CAH.
Gomes, L.G. 2020 (35)	Review Paper	Pediatric and Adult	Several studies showed increased prevalence of obesity, abnormal body composition, insulin resistance, and hypertension in CAH patients.	Despite an increased prevalence of cardiovascular markers, CVD remains unknown, and comparison of varying glucocorticoid regimens is needed.
Paizoni, L. 2020 (36)	N = 90	18-62 years	IMT was the same between CAH patients and controls. Only one patient in the cohort fulfilled the criteria for metabolic syndrome.	Though there is a high prevalence of insulin resistance and obesity in CAH patients, rarely do adults with CAH develop metabolic syndrome.
		(Median 29y)		
		57% Female		
Farghaly, H.S. 2021 (37)	N = 40	14.8 ± 2.6 years	CAH patients had elevated serum neopterin levels, decreased brachial FMD %, and normal cIMT vs. controls.	CAH patients have endothelial dysfunction as noted by elevated serum neopterin levels, which can explain vascular pathology seen in CAH.
		70% Female		
Hasemi Dehkordi, E. 2021 (38)	N = 78	9.40 ± 4.09 years	17-OHP serum concentrations were positively correlated with DBP and BMI in CAH patients.	Elevated 17-OHP, a marker of poor disease management, may be correlated to increased prevalence of CVD risk factors in CAH patients.
		53% Female		
Torky, A. 2021 (14)	N = 57	Pediatric andAdult (longitudinal)	CAH patients exhibited a higher prevalence of obesity, hypertension, insulin resistance, and low HDL that began prior to age 10. 23 patients fit metabolic syndrome criteria at 1+ visits. Increased obesity in childhood was seen with maternal obesity.	Higher prevalence of CVD risk factors is seen in CAH patients at a young age and is associated with treatment and familial factors.

## Traditional Cardiometabolic Risk Factors

### Obesity

Compared to unaffected controls, youth with CAH present a higher prevalence of obesity overall, with one large study finding the median age of onset to be 8 years old (14). Not only is obesity more prevalent in youth with CAH, but a centralized fat distribution and increased waist-to-height ratio have also been observed, suggesting a more unfavorable distribution of body fat (17, 22, 27, 35). In youth with CAH, the fat mass-to-lean mass ratio was also recently shown to be significantly higher compared to controls (36). Central obesity or an ‘apple-shape’ indicates increased abdominal adipose tissue, which is of particular concern as visceral abdominal adipose tissue (VAT) is highly proinflammatory in individuals with obesity and metabolic syndrome (39). Youth with CAH exhibit increased VAT and subcutaneous adipose tissue (SAT) compared to BMI-matched controls, with an increased VAT-to-SAT ratio compared to controls as well, which constitutes an adverse metabolic phenotype in obese adolescents (15).

In addition to fat distribution, youth with CAH also exhibit an earlier age at adiposity rebound compared to their unaffected peers. Adiposity rebound (AR) is known as the second rise in BMI during childhood that corresponds to an increase in number of adipocytes (40, 41). In normative populations, AR takes place between 5 and 7 years old; however, for youth with CAH, age at AR has been shown in the U.S. and Japan to occur at approximately 3 years (7, 26, 34), with children in the U.K. exhibiting an even earlier AR at 1.7 years old (4). Youth with CAH and obesity had an even earlier age at AR in the U.S., at 2.8 years, with an earlier AR predicting a higher BMI z-score and central obesity in later childhood (34). Thus, early AR in patients with CAH could help identify youth at risk for cardiometabolic disease.

As well, familial factors such as maternal obesity during childhood can contribute to the increased incidence of obesity seen in this cohort (14).

### Hypertension

High blood pressure is another major risk factor for cardiovascular disease, and in CAH youth, there is an increased frequency of hypertension overall observed across age groups, although more prevalent in younger children compared to adolescents (11). Youth with CAH exhibit higher systolic blood pressure compared to controls (30, 31) and have been shown again recently to exhibit an impaired or absent nocturnal drop in blood pressure compared to controls (21, 36).

Hypertension has been found to occur more frequently in patients with CAH who receive fludrocortisone therapy compared to those who are not taking fludrocortisone (42). There has also been some recent evidence that the negative correlation between blood pressure and age could be explained by an overall reduction in fludrocortisone dose as patients with CAH become older (31). Suppressed plasma renin activity levels have also been shown to be correlated with high blood pressure (5, 14, 35).

In terms of contributing factors, among youth with CAH there is a positive correlation between BMI and blood pressure (27, 33), suggesting a meaningful relationship between prevalence of obesity and hypertension in this population. As well, 17-hydroxyprogesterone (17-OHP) levels have also been noted to be positively correlated with diastolic blood pressure and BMI (38). Conversely, a higher 17-OHP has been found to be protective against hypertension in a large study of children, while suppressed androstenedione was noted to be associated with hypertensive BP, perhaps indirectly representing an effect of excess glucocorticoid dosing on blood pressure (14).

Finally, sexual dimorphism has been noted in pubertal adolescents ages 12-18 years old, with high blood pressure found to be more prevalent in females compared to males with CAH (11).

### Elevated Fasting Blood Glucose

Recent studies have shown that patients with CAH may exhibit a higher prevalence of fasting hyperglycemia during childhood compared to controls (14, 29). In a large longitudinal study of patients with CAH, the prevalence of elevated fasting blood glucose was shown to increase during school age and adolescence, but to decrease in young adulthood (14). Elevated fasting plasma glucose levels have been observed in adult patients with CAH (43), with emerging reports that this also may occur during childhood; however, it has been more common to see insulin resistance than hyperglycemia reported in youth with CAH.

### Dyslipidemia

There has been a relatively small number of studies reporting dyslipidemia in youth with CAH, with higher triglycerides, lower HDL cholesterol, and higher small dense-LDL having recently been reported (14, 31). Nonetheless, dyslipidemia in youth with CAH has been shown to worsen with age, in particular the prevalence of low HDL in adulthood (14). Elevated levels of the androgen precursor, 17-OHP, used as a marker of disease severity and/or hormonal control, appear to negatively correlate with incidence of hypercholesterolemia and are associated with low HDL levels (14). Worse hormonal control (higher 17-OHP) could be relatively protective for dyslipidemia if those patients exhibiting tighter hormonal control (*i.e.*, lower 17-OHP) are therefore on higher glucocorticoid replacement (14). Overall, the evidence supporting an increased risk of dyslipidemia in youth with CAH has been variable.

## Non-Traditional Cardiometabolic Risk Factors

### Subclinical Atherosclerosis

Both flow-mediated dilation (FMD) of the brachial artery and intima-media thickness of the carotid artery (cIMT) are early surrogate markers of atherosclerosis that have been studied in youth with CAH. Vascular endothelial and smooth muscle dysfunction, as measured by a decreased FMD, has been shown in youth with CAH (17, 37) even after correcting for age, sex, BMI, and doses of glucocorticoid and fludrocortisone (28). Although endothelial dysfunction is a critical early step in the development of atherosclerosis and can serve as a potential predictor of cIMT (44), there have been mixed results in youth and young adults with CAH in terms of group differences in cIMT compared to controls (16, 17, 23, 24, 32, 45). Among youth with CAH, however, cIMT has been positively correlated with androgen levels (24, 29).

Additionally, markers for endothelial dysfunction have been studied such as neopterin, a novel inflammatory biomarker for endothelial damage that has been notably elevated in patients with CAH (37, 46–48). Elevated high-sensitivity C-reactive protein (hs-CRP) and circulating endothelial cell levels in serum are also seen in youth with CAH, suggesting endothelial damage and subclinical atherosclerosis (25).

Epicardial fat thickness is another emerging early marker of atherosclerosis and has also been noted to be higher in youth with CAH (32). Epicardial fat thickness was also positively correlated with waist circumference, 17-OHP, and insulin resistance, suggesting relationships with other cardiometabolic risk factors (32).

Further study is merited to understand the contribution of increased vascular endothelial injury and endothelial dysfunction to the development of higher blood pressure seen in youth with CAH.

### Inflammatory Markers

Youth with CAH exhibit increased circulating concentrations of inflammatory markers compared to unaffected youth, which is important given they are surrogate markers of future cardiovascular disease (49). There could be several reasons for increased inflammation in youth with CAH, including increased VAT which produces more inflammatory substances associated with cardiovascular disease, promotes inflammation in the body, and is associated with risk for metabolic disease independent of total body adiposity (50). Youth with CAH have been shown to have significantly higher leptin concentrations compared to controls (15, 19, 31), potentially caused by epinephrine deficiency (19), and/or an altered leptin axis related to decreased soluble leptin receptor (51). Leptin levels are also positively correlated with obesity (27, 31) and abdominal fat (15) in youth with CAH. The inflammatory markers, PAI-1 and hs-CRP were correlated with abdominal fat as well (15). Lastly, homocysteine levels, an inflammatory marker for atherosclerosis and coronary artery disease, have also been shown to be increased in patients with CAH (29).

### Insulin Resistance

In youth with CAH, a higher prevalence of insulin resistance has been found compared to their unaffected peers, with significantly higher insulin concentrations and homeostasis model assessment for insulin resistance index (HOMA-IR), even after adjusting for BMI (14, 22, 32). The prevalence of insulin resistance in youth with CAH increases with age (14). Among youth with CAH, insulin resistance has been related to hydrocortisone dose, BMI-SDS, and plasma renin activity levels, but not with hyperandrogenism (12, 35). Some suggest that lower hydrocortisone doses could lead to a reduction of insulin resistance (8); however, this may only be true when the doses are supraphysiologic (14). Although there is increased insulin resistance and fasting hyperglycemia in youth with CAH, there has not been an increase in type 2 diabetes yet noted (35).

## Discussion

Traditional cardiometabolic risk factors may occur in youth with classical CAH due to 21-hydroxylase deficiency and continue to be present throughout childhood, although the metabolic syndrome itself has not been as commonly reported as might be expected. However, a longitudinal natural history study recently identified 23 cases of metabolic syndrome with a median age of onset of 9.6 years (14). A higher prevalence of obesity during childhood and adulthood, along with hormone replacements over the lifetime, could be contributing factors for hypertension and insulin resistance across all ages. Combined with emerging reports of type 2 diabetes, gestational diabetes, and adverse cardiac events in adults with classical CAH (20, 52, 53), further longitudinal study of this high-risk cohort is merited to assess risk factors from childhood through adulthood, to better understand the development of longer-term adverse outcomes. As well, the examination of non-traditional cardiometabolic risk factors as potential early biomarkers for subclinical atherosclerosis, inflammation, and insulin resistance could be useful in patients with CAH.

The pathophysiology underlying the increased prevalence of cardiometabolic risk factors in patients with CAH is not yet fully understood. However, both disease- and treatment-related factors should be considered. Decreased cortisol production in CAH necessitates lifelong glucocorticoid replacement, with studies pointing to the *supraphysiologic* glucocorticoid doses needed to suppress excess ACTH signaling to the adrenal gland, as contributing to the development of cardiometabolic risk factors (1, 31). The management of hyperandrogenism in patients with CAH is often a challenge with many patients having persistent elevations in circulating androgens despite attempts at optimization of glucocorticoid dosing, although there may be a trade-off between hormonal control and glucocorticoid dosing, in terms of cardiometabolic risk (1, 14). Multiple adjunctive therapies are currently under investigation, including CRH and corticotropin-releasing factor receptor antagonists, along with extended-release formulations of hydrocortisone, to decrease overall daily glucocorticoid dosing in patients with the goal of minimizing side effects related to the currently used supraphysiologic doses of glucocorticoid. As well, mineralocorticoid supplementation may potentially add to risk for hypertension and lipid abnormalities in youth with CAH.

Disease-related contributing factors to consider in youth with classical CAH include hyperandrogenism and adrenomedullary dysfunction. The contribution of hyperandrogenism is complicated to assess in patients with CAH who are already on hormone replacement therapies, presenting a need for more biomarkers with which to study prenatal and cumulative androgen exposure over the lifetime in these patients (54). We know that hyperandrogenic females with polycystic ovarian syndrome (PCOS) exhibit the traditional and non-traditional cardiometabolic risk factors that are present in patients with CAH and can occur at an early age (55–58). In addition, transgender men utilizing gender-affirming testosterone therapy are another important cohort that is chronically exposed to androgens and can exhibit an increase in BMI, dyslipidemia, and vascular dysfunction (59–61). Androgen exposure in females is associated with endothelial dysfunction and can directly contribute to vascular dysfunction and high blood pressure (60, 62). It should be noted though that these related natural human models of hyperandrogenism do not involve the additional inherent hormone imbalances found in patients with classical CAH (*e.g.*, lower cortisol, aldosterone, and epinephrine production). Patients with classical CAH have an additional deficiency in epinephrine (19, 63–65), which could lead to a lack of stimulated lipolysis of triglyceride stores, and dysregulation of insulin and adipokines (19). It is also interesting to consider the implications of lower epinephrine levels and disturbed adrenomedullary function under fasting and feeding conditions that have been noted in unaffected adults with obesity (66). Future research is needed to study the role of adrenomedullary dysfunction in the pathogenesis of cardiometabolic risk in patients with CAH.

Given the early onset of associated cardiometabolic risk factors, and prolonged hormone imbalances already present *in utero*, the assessment of children with CAH from an early age is merited to better understand prenatal and early postnatal origins of cardiometabolic disease in patients with CAH. Key components of the metabolic syndrome, *i.e.*, obesity and hypertension, can arise early in childhood in patients with CAH, with obesity itself linked to the development of hypertension, insulin resistance, type 2 diabetes, dyslipidemia, and long-term vascular complications. In patients with CAH, obesity is associated with the development of cardiometabolic risk factors in adults (14). Therefore, it will be particularly important to further understand the mechanism driving the increased prevalence of obesity in children with CAH and to provide appropriate interventions at an early age. While healthy lifestyle counseling should commence early in childhood as part of routine clinical practice guidelines (67), the development of medical therapeutics to treat obesity and insulin resistance in these high-risk youth with CAH may also be useful to prevent cardiometabolic sequelae and metabolic syndrome in adulthood.

## Conclusions and Perspectives

There is a need for the longitudinal study of patients with classical CAH from diagnosis at infancy through older adulthood to better characterize the natural history of the metabolic syndrome and its components, along with cardiovascular disease. While there is an underlying relationship between treatment-related factors and cardiometabolic risk factors, more needs to be understood about the contribution of disease-related factors in CAH amidst the challenges of studying a cohort on hormone replacement from an early age. Gaining a better understanding of both traditional and non-traditional risk factors and their effects on youth with CAH could ultimately lead to the improved treatment and prevention of metabolic syndrome and cardiovascular disease in adulthood.

## Author Contributions

MK, NF, and NM performed an extensive literature search and drafted the manuscript. All authors critically reviewed the manuscript. All authors contributed to the article and approved the submitted version.

## Funding

The writing of this manuscript was supported by NIH grants, K23HD084735 and R03HD101718 (NIH/NICHD to MK), Abell Foundation and Grace Nixon Foundation (to MG), and a Keck Summer Research Fellowship (to NM). We thank CARES Foundation for support of the CAH Comprehensive Care Center at CHLA.

## Author Disclaimer

The contents of this work are solely the responsibility of the authors and do not necessarily represent the official views of the National Institutes of Health.

## Conflict of Interest

MG receives research support from Novo Nordisk, Adrenas Therapeutics, Neurocrine Biosciences, and Spruce Biosciences. MG serves on advisory boards or as a consultant for Adrenas Therapeutics, Ascendis, Eton Pharmaceuticals, Novo Nordisk, and Pfizer; serves on data safety monitoring boards for Ascendis and Saniona/Medpace; serves as an adjudication committee member for ICON Clinical Research, LLC/Aeterna Zentaris; and receives royalties from McGraw-Hill and UpToDate. MK receives research support from Neurocrine Biosciences and Spruce Biosciences.

The remaining authors declare that the research was conducted in the absence of any commercial or financial relationships that could be construed as a potential conflict of interest.

## Publisher’s Note

All claims expressed in this article are solely those of the authors and do not necessarily represent those of their affiliated organizations, or those of the publisher, the editors and the reviewers. Any product that may be evaluated in this article, or claim that may be made by its manufacturer, is not guaranteed or endorsed by the publisher.
